# The use of omics technologies in creating LBP and postbiotics based on the *Limosilactobacillus fermentum* U-21

**DOI:** 10.3389/fmicb.2024.1416688

**Published:** 2024-06-11

**Authors:** Maya V. Odorskaya, Dilara A. Mavletova, Andrey A. Nesterov, Olga V. Tikhonova, Natalia A. Soloveva, Diana A. Reznikova, Olesya O. Galanova, Aleksey A. Vatlin, Nikolai M. Slynko, Asya R. Vasilieva, Sergey E. Peltek, Valery N. Danilenko

**Affiliations:** ^1^Laboratory of Bacterial Genetics, Vavilov Institute of General Genetics Russian Academy of Sciences, Moscow, Russia; ^2^Institute of Environmental Engineering, RUDN University, Moscow, Russia; ^3^Institute of Biomedical Chemistry, Moscow, Russia; ^4^Phystech School of Biological and Medical Physics, Moscow Institute of Physics and Technology, Dolgoprudny, Russia; ^5^Institute of Cytology and Genetics, Siberian Branch of Russian Academy of Sciences, Novosibirsk, Russia; ^6^Brain Science Institute, Research Center of Neurology, Moscow, Russia

**Keywords:** pharmacobiotics, postbiotics, *Limosilactobacillus fermentum* U-21, metabolome, proteome, omics technologies

## Abstract

In recent years, there has been an increasing tendency to create drugs based on certain commensal bacteria of the human microbiota and their ingredients, primarily focusing on live biotherapeutics (LBPs) and postbiotics. The creation of such drugs, termed pharmacobiotics, necessitates an understanding of their mechanisms of action and the identification of pharmacologically active ingredients that determine their target properties. Typically, these are complexes of biologically active substances synthesized by specific strains, promoted as LBPs or postbiotics (including vesicles): proteins, enzymes, low molecular weight metabolites, small RNAs, etc. This study employs omics technologies, including genomics, proteomics, and metabolomics, to explore the potential of *Limosilactobacillus fermentum* U-21 for innovative LBP and postbiotic formulations targeting neuroinflammatory processes. Proteomic techniques identified and quantified proteins expressed by *L. fermentum* U-21, highlighting their functional attributes and potential applications. Key identified proteins include ATP-dependent Clp protease (ClpL), chaperone protein DnaK, protein GrpE, thioredoxin reductase, LysM peptidoglycan-binding domain-containing protein, and NlpC/P60 domain-containing protein, which have roles in disaggregase, antioxidant, and immunomodulatory activities. Metabolomic analysis provided insights into small-molecule metabolites produced during fermentation, revealing compounds with anti-neuroinflammatory activity. Significant metabolites produced by *L. fermentum* U-21 include GABA (γ-aminobutyric acid), niacin, aucubin, and scyllo-inositol. GABA was found to stabilize neuronal activity, potentially counteracting neurodegenerative processes. Niacin, essential for optimal nervous system function, was detected in vesicles and culture fluid, and it modulates cytokine production, maintaining immune homeostasis. Aucubin, an iridoid glycoside usually secreted by plants, was identified as having antioxidant properties, addressing issues of bioavailability for therapeutic use. Scyllo-inositol, identified in vesicles, acts as a chemical chaperone, reducing abnormal protein clumps linked to neurodegenerative diseases. These findings demonstrate the capability of *L. fermentum* U-21 to produce bioactive substances that could be harnessed in the development of pharmacobiotics for neurodegenerative diseases, contributing to their immunomodulatory, anti-neuroinflammatory, and neuromodulatory activities. Data of the HPLC-MS/MS analysis are available via ProteomeXchange with identifier PXD050857.

## 1 Introduction

Comprehensive studies of the human intestinal microbiome under normal and pathological conditions have made it possible to identify specific genera and species of commensal bacteria responsible for maintaining healthy homeostasis of the body (Azad et al., 2018; Ueda et al., 2021). A number of biomarkers (metabolites) have been identified that are responsible for the neuromodulatory, immunomodulatory and antioxidant potential of the human intestinal microbiota (Kovtun et al., 2018, 2022; Props et al., 2019; Averina et al., 2020, 2021; Klann et al., 2021; Donati Zeppa et al., 2022; Morton et al., 2023). There is an active search for drug candidates among intestinal commensal bacteria (not only lacto- and bifidobacteria) (Mo et al., 2022; Mohebali et al., 2023; Murali and Mansell, 2024; Seo et al., 2024). Whole-genome sequencing of many hundreds of classical probiotic strains has been carried out. Methods of proteomic and metabolomic analysis are actively used to characterize probiotic strains of *Lactobacilli* (Ming et al., 2018; Xu et al., 2021; Chamberlain et al., 2022).

In recent years, alongside the development of probiotics, there has been a growing trend towards creating LBP and postbiotics. According to FDA guidelines (June 2016), a drug can be classified as a live biotherapeutic product if it is developed from living bacteria, has undergone preclinical and clinical trials, and is used to prevent or treat a specific disease. Essentially, it is proposed to study it according to the scheme used in the development of most drugs, taking into account the specificity that it is a complex of pharmacologically active ingredients synthesized by a specific bacterium. Taking into account the development of LBP for the treatment or prevention of specific diseases, for example, neurodegenerative ones, at the first stage arises the task of selecting a specific strain of a certain species of bacteria capable of synthesizing a complex of substances with specified properties. This can be carried out in complex studies of a given strain using genomic and omics technologies. At a certain stage, the option of creating postbiotic products based on LBP (Ağagündüz et al., 2022), including extracellular vesicles.

Postbiotics are metabolites or components produced by the microbiota that significantly affect human health and have a proven mechanism of action (Salminen et al., 2021; Thorakkattu et al., 2022). Postbiotics are mainly associated with immunomodulatory anti-inflammatory activity, playing a role in maintaining the integrity of the intestinal mucosal barrier and counteracting pathogens with antimicrobial compounds by stimulating the innate and adaptive immune system (De Marco et al., 2018; Singh et al., 2018).

Recently, the definition of postbiotics has been broadened to include vesicles formed by inhabitants of the gut microbiome (Domínguez Rubio et al., 2022). Some *Lactobacillus* produce extracellular vesicles, spherical bilipid membrane structures, ranging in size from 20 to 200 nm in the culture medium (Dean et al., 2019). Vesicles are thought to contain various proteins, enzymes, RNA and metabolites (Briaud and Carroll, 2020). Extracellular vesicles have a function not only in bacterial intercellular communication, but can also pass through cell membranes into eukaryotic cells, transporting various substances (Jang et al., 2015). Vesicles of intestinal bacteria enter the bloodstream through the intestinal wall (Stentz et al., 2018). Extracellular vesicles of *Lactobacillus* spp. have beneficial effects on the host by modulating the host immune system (Domínguez Rubio et al., 2017; Kim et al., 2018; Dean et al., 2019; González-Lozano et al., 2022), so they are considered as a potential postbiotic drug.

*Lactobacillus* is a family of the gut microbiome that is a natural source of postbiotics due to its ability to synthesize bioactive compounds and interact with the host organism. Because of this, it modulates the immune status and antioxidant (AO) status of the host (Nowak et al., 2019; Poluektova et al., 2021; Zhou et al., 2022). The antioxidant properties of many *Lactobacillus* species have already been confirmed by many *in vivo* and *in vitro* studies (Marsova et al., 2018, 2020; Noureen et al., 2019; Averina et al., 2021).

The *Limosilactobacillus fermentum* U-21 strain used in this study and first obtained in the Laboratory of Bacterial Genetics, Vavilov Institute of General Genetics Russian Academy of Sciences, is a potential LBP and postbiotic source. The strain as a subject of the study was chosen earlier on the basis of its ability to reduce oxidative stress under the action of superoxide anion in a bioluminescent test system based on *Escherichia coli* MG1655 strain carrying plasmids encoding luminescent biosensors pSoxS-lux and pKatG-lux (Marsova et al., 2018). The antioxidant properties of the strain were later confirmed using *in vivo* and *in vitro* models. In particular, *L.fermentum* U-21 prolonged the life of *Caenorhabditis elegans* nematode by 25% under paraquat-induced oxidative stress (Marsova et al., 2020). In a model of induced Parkinsonism in mice, *L.fermentum* U-21 prevented degradation of brain dopaminergic neurons and pathologic changes in internal organs (Marsova et al., 2020; Stavrovskaya et al., 2024). Genomic analysis of *L.fermentum* U-21 strain identified 29 genes associated with antioxidant potential, the most important of which are genes of thioredoxin complex, metabolism and transport of heavy metals (Poluektova et al., 2022).

The aim of this study is to develop a technology for the production of LBP and postbiotics using omics technologies. Omics technologies are based on the achievements of genomics, proteomics, metabolomics. These sciences study how genome is arranged and how the information encoded in it is realized, how this information is transformed into structure of proteins and further into some features of the organism. All this may be important for diagnostics and treatment of diseases. Omics technologies are one of the main tools of genomic and post-genomic medicine. To achieve the goal, the following objectives were set: comparative analysis (proteomic, metabolomic) of two *L.fermentum* strains with high and low antioxidant potentials, establishing the distribution of potential pharmacologically active metabolites in living cells and postbiotics (culture fluid and vesicles), integrative analysis of proteomic and metabolomic data on targeting indicators: antioxidation, anti-inflammatory and immunomodulatory, consider the possibility of using *L.fermentum* U-21 and postbiotics based on it to relieve inflammatory processes and application for the prevention and treatment of neurodegenerative diseases.

## 2 Materials and methods

### 2.1 Bacterial strains

In this study, we used *L.fermentum* U-21 and *L.fermentum* 279, both of which were isolated from the organisms of people who lived in the Central European region of the Russian Federation. Both strains are stored at the Research Topic of the laboratory of genetics of microorganisms, Vavilov Institute of General Genetics Russian Academy of Sciences. The genomes of *L.fermentum* U-21 and 279 strains were sequenced and annotated (Table 1).

**Table 1:** Genetic characterization of strains.

Strain	BioSample ID	GenBank Sequence
*LimosiLactobacillus fermentum* U-21	SAMN08290293	GCA_002869825.2
*LimosiLactobacillus fermentum* 279	SAMN08014151	GCA_002794275.1

### 2.2 Method, conditions, and media for strain propagation

The strains *L.fermentum* U-21 and *L.fermentum* 279 were both grown on the MRS media (Himedia) under partially anaerobic conditions. The MRS media contained 10.0 g/L proteosopeptone, 10.0 g/L meat extract, 5.0 g/L yeast extract, 20.0 g/L glucose, 1.0 g/L polysorbate 80, 2.0 g/L ammonium citrate, 5.0 g/L sodium acetate, 0.1 g/L magnesium sulfate, 0.05 g/L manganese sulfate, and 2.0 g/L dibasic potassium phosphate (pH 6.5 at 25°C). The cultivation temperature was 37°C.

### 2.3 Sample preparation for proteomic analysis

Bacterial cells were separated from the culture liquid by centrifugation at 7,000*g* for 30 min at 4°C, then the culture liquid was filtered through a PES membrane (0.22 μm). The cell samples were lysed using ice-cold buffer (150 μL) containing 5% SDS with subsequent ultrasonication using the Bandelin Sonopuls probe (“BANDELIN electronic GmbH & Co. KG,” Berlin, Germany). The sample protein concentration was measured using a Pierce™ BCA Protein Assay Kit (Pierce, Rockford, IL, United States). Trypsin digestion was then performed according to the S-Trap sample preparation method according to the manufacturer’s manual (Profity, Fairport, NY, United States). The obtained peptide concentrations were determined by the colorimetric method using a Pierce™ Quantitative Colorimetric Peptide Assay kit (Thermo Scientific, Waltham, MA, United States) in accordance with the manufacturer’s recommendations.

### 2.4 Extracellular vesicles preparation for proteome and metabolome analysis

Bacterial cells were separated from the culture liquid at 7,000*g* for 10 min at 4°C. After centrifugation, the culture liquids were filtered through a PES membrane (0.22 μm). 250 mL of supernatant was used to isolate the extracellular vesicles using ultracentrifugation at 260,000*g* for 1 h 40 min at 4°C and resuspended in Phosphate-buffered saline (PBS, pH 7.4). The resulting suspension was filtered through a PES filter (0.45 μm) and ultracentrifuged under the same conditions. Using a Qubit 3.0 fluorometer, the protein quantities in extracellular vesicles were quantified (Life Technologies, Grand Island, NY).

### 2.5 HPLC-MS/MS analysis

The HPLC-MS/MS was performed at the ‘Human Proteome’ core facility center of the Institute of Biomedical Chemistry (Moscow, Russia).

One microgram of peptides in a volume of 1–4 μL was loaded onto the Acclaim μ-Precolumn (0.5 mm × 3 mm, 5 μm particle size, Thermo Scientific) at a flow rate of 10 μL/min for 4 min in an isocratic mode of Mobile Phase C (2% acetonitrile, 0.1% formic acid). Then the peptides were separated with high-performance liquid chromatography (HPLC, Ultimate 3,000 Nano LC System, Thermo Scientific, Rockwell, IL, United States) in a 20-cm long C18 column (Peaky, inner diameter of 100 μm, Molecta, Russia). The peptides were eluted with a gradient of buffer B (80% acetonitrile, 0.1% formic acid) at a flow rate of 0.3 μL/min. Total run time was 90 min, which included initial 4 min of column equilibration to buffer A (0.1% formic acid), then gradient from 5 to 40% of buffer B over 65 min, then 6 min to reach 99% of buffer B, flushing 10 min with 99% of buffer B and 5 min re-equilibration to buffer A.

MS analysis was performed at least in triplicate with a Q Exactive HF mass spectrometer (Q Exactive HF Hybrid Quadrupole-OrbitrapTM Mass spectrometer, Thermo Fisher Scientific, Rockwell, IL, United States). The temperature of capillary was 240°C and the voltage at the emitter was 2.1 kV. Mass spectra were acquired at a resolution of 120,000 (MS) in a range of 390–1,500 m/z. Tandem mass spectra of fragments were acquired at a resolution of 60,000 (MS/MS) in the range from 120 m/z to m/z value determined by a charge state of the precursor. The maximum integration time was 50 ms and 110 ms for precursor and fragment ions, correspondently. AGC target for precursor and fragment ions were set to 1 × 10^6^ and 1 × 10^5^, correspondently. An isolation intensity threshold of 400,000 counts was determined for precursor’s selection and up to top 20 precursors were chosen for fragmentation with high-energy collisional dissociation (HCD) at 29 NCE. Precursors with a charge state of +1 and more than +5 were rejected and all measured precursors were dynamically excluded from triggering of a subsequent MS/MS for 40 s.

### 2.6 Protein identification

Raw MS data files were analyzed using the MaxQuant search engine (v.2.0.3.0) with the build-in Andromeda algorithm (Tyanova et al., 2016). The UniProt FASTA database for *Limosilactobacillus fermentum* (June 2022) concatenated with a reverse decoy database was used for proteins identification. Trypsin was specified as cleavage enzyme allowing up to two missing cleavages. The mass tolerance for precursor ions was set as 20 ppm in First search and 5 ppm in Main search, respectively, Carbamidomethyl of Cys was specified as fixed modification and oxidation on Met was specified as variable modifications. “Match between runs” option have been also applied for technical replicates.

### 2.7 Culture fluids preparation for metabolome analysis

For metabolomic analysis, the bacterial cultures were grown to the stationary growth phase (OD600 = 2.5). Bacterial cells were separated from the culture liquid by centrifugation at 7,000*g* for 10 min at 4°C. After centrifugation, the culture liquids were filtered through a PES membrane (0.22 μm). Filtered culture fluid was evaporated using RE-52AA Rotary Evaporator (HEB Biotechnology, China).

### 2.8 Extraction of metabolites

The metabolites were extracted with a mixture of isopropanol, acetonitrile and water (3:3:2, v/v/v). An aliquot was evaporated until it completely dried out, and then redissolved in a mixture of acetonitrile and water (1:1, v/v); the supernatant was evaporated until it completely dried out.

### 2.9 Derivatization of metabolites

10 μL of 20 mg/mL methoxyamine pyridine hydrochloride was added for derivatization, and the mixture was then shaken vigorously for 1.5 h at 30°C. The samples were then further derivatized by adding 91 μL of a mixture of MSTFA and FAME to each sample, which was then incubated at 37°C for 30 min on a thermal shaker.

### 2.10 GC × GC–MS analysis

In two-dimensional mode, the samples were analyzed on a Pegasus 4D GC × GC–TOF MS instrument with the following settings: injection, 1 mL; pulsed split, 1:100, 250°C; carrier gas (He) flow, 1.4 mL/min, corrected constant flow; column one, Rxi-5МS, 30 m × 0.25 mm i.d. × 0.25 μm coating (Restek); column two, Rxi-17Sil МS, 1.75 m × 0.25 mm i.d. × 0.25 μm coating (Restek); temperature program, 50°C (1 min), then at 5°C/min to 150°C, at 10°C/min to 250°C, and at 20°C/min to 280°C (and held there for 60 min); primary oven was kept at 5°C higher than secondary oven; modulation, 8 s with temp. Maintained at 15°C above secondary oven; transfer line, 280°C; ion source temp., 280°C; mass range (m/z), 40–850 (Vasilieva et al., 2023).

### 2.11 Data analysis

The resulting spectrum files were processed in ChromaTOF (v. 5.51, LECO, United States) for deconvolution, peak selection, alignment, and search in the primary database. Metabolites were identified based on mass spectra and retention times from the National Institute of Standards and Technology (NIST) libraries, the Mainlib and Feign libraries, and the National Institutes of Health (NIH) public repository.

We used ChromaTOF Tile v.1.01 (LECO, United States) to reduce the multidimensionality of experimental data based on the Fisher coefficient and identify significantly different chemicals in the culture liquids. The processing principle of ChromaTOF Tile v.1.01 is the comparison of two matching sections of the chromatogram (so-called tiles) and highlighting the low and high levels. The size of the studied cells was 3 × 24 in modulation and spectral measurements, respectively. Only results with a signal-to-noise ratio greater than 70 were counted. The range of analyzed masses was limited from m/z = 85 to m/z = 700. Identification was performed using the NIST database for mass spectra and retention indices (mainlib, replib) and the Leco-Fiehn rtx5 library. The matches with a direct and reverse similarity of more than 700 were considered significant. All component’s concentration values are calculated from the peak areas in chromatograms through the peak area of caffeine and are expressed in μg per gram of the corresponding fraction.

### 2.12 THP-1 cell line and cultivation conditions

The THP-1 cell line was cultivated in RPMI-1640 medium (PanEco, Russia) with 10% fetal bovine serum (FBS) (PanEco, Russia), 292 mg/L L-glutamine (PanEco, Russia), 25,000 units of penicillin and 25,000 mcg of streptomycin. The cell line was cultivated in culture flasks (with a cell growth surface of 25 cm^2^), maintaining a culture density of 10^6^–10^7^ cells/mL in a sterile incubator under optimal conditions—37°C and 5% CO2. For immunomodulatory activity estimation cells were stimulated with bacterial samples (*L.fermentum* U-21 bacteria cells, *L.fermentum* 279 bacteria cells, *L.fermentum* U-21’s culture fluid and vesicles) and/or LPS (*E. coli* 0127:B8 lipopolysaccharide, Sig-ma-Aldrich, United States) at a concentration of 1,000 ng/mL for 3 h. Bacteria were added at 50 bacteria per cell, supernatant at 1.5 mL, and vesicles at 70 mg of protein.

Preliminary for the experiment, *L.fermentum* U-21 and *L.fermentum* 279 strains were cultivated under partially an-aerobic conditions at 37°C in MRS Broth medium until a culture density of 5 × 10^7^ CFU/mL (OD600 = 0.8) was reached, then centrifuged for 10 min at 14,000*g*. The resulting supernatant (cultural fluid) was filtered through a 0.22 μm PES filter, and the pH of the culture medium was adjusted to 7.0 with NaOH solution (10 M). The bacteria cells were washed with RPMI-1640 medium to culture with THP-1 cells.

### 2.13 Isolation and purification of RNA from THP-1 cells, cDNA synthesis

To isolate the total RNA of the THP-1 cell line, ExtractRNA (Evrogen, Russia) was used following the manufacturer’s protocol. The remaining genomic DNA was removed by DNAse I, Amplification grade (Invitrogen, United States). A volume of 50 ng of total RNA was used for cDNA synthesis by iScript Select cDNA Synthesis Kit (Bio-Rad, Berkeley, CA, United States). A sample of 1 ng of cDNA was used for real-time qPCR with the qPCRmix-HS SYBR kit (Evrogen, Russia) on a CFX96 Touch machine (Bio-Rad, United States). The CFX Manager V 3.1 (Bio-Rad, United States) was used to analyze the qPCR results: relative normalized expression of three biological replicates was calculated as ΔΔCq and the gene *gapdh* was used as a reference (‘Demonstration of a ΔΔCq Calculation Method to Compute Thermo Scientific Relative Gene Expression from qPCR Data | SelectScience’, n.d.). The primers were picked by primer-BLAST for qPCR (Supplementary Table S1) (Ye et al., 2012).

### 2.14 The search for metabolic pathways in *L.fermentum* U-21

#### 2.14.1 Aucubin

A metabolic pathway of terpenoid biosynthesis was discovered for the *LimosiLactobacillus fermentum* strain IFO 3956.^1^ The homologous proteins’ genes of this metabolic pathway were searched for in the genome of the *L.fermentum* strain U-21 using blast+. All genes responsible for the synthesis of enzymes in the terpenoid biosynthesis pathway (from acetyl-CoA to geranyl pyrophosphate) in the *L.fermentum* U-21 strain were found. The homology with amino acid sequences of the strain *L.fermentum* IFO 3956 was more than 90%. The next step was searching for the metabolic pathway of monoterpenoids including aucubin in *L.fermentum* U-21. Nowadays this pathway is not fully described. *Catharanthus roseus*’ enzymes have been selected for the search. Geranyl pyrophosphatase was found in *M.tuberculosis* H37Rv (swiss-prot). First alignment was carried out for the genome of the *L.fermentum* IFO 3956, as terpenoid biosynthesis had already been detected in it and entered into the KEGG Pathway database. Among the obtained sequences, the most suitable ones were selected according to a number of criteria: (i) the length of the matching section is at least 200 a.a. (ii) homology of at least 30% with comparison proteins (iii) similar protein domains. According to the selected amino acid sequences one more alignment was carried out for the *L.fermentum* U-21 strain.

#### 2.14.2 Niacin

The metabolic pathway of niacin is described for some Firmicutes. Amino acid sequences were selected among Firmicutes in swiss-prot for searching genes of homologous proteins in the *L.fermentum* U-21. The metabolic pathway of niacin is described in KEGG Pathway.^2^ Next alignment was carried out for the selected sequences. The results were selected in accordance with the following requirements: (i) sequence length match >90%; (ii) protein homology >60%.

## 3 Results

### 3.1 Comparison of GC–MS metabolite profiles of *L.fermentum* U-21 and *L.fermentum* 279

**Figure 1:**
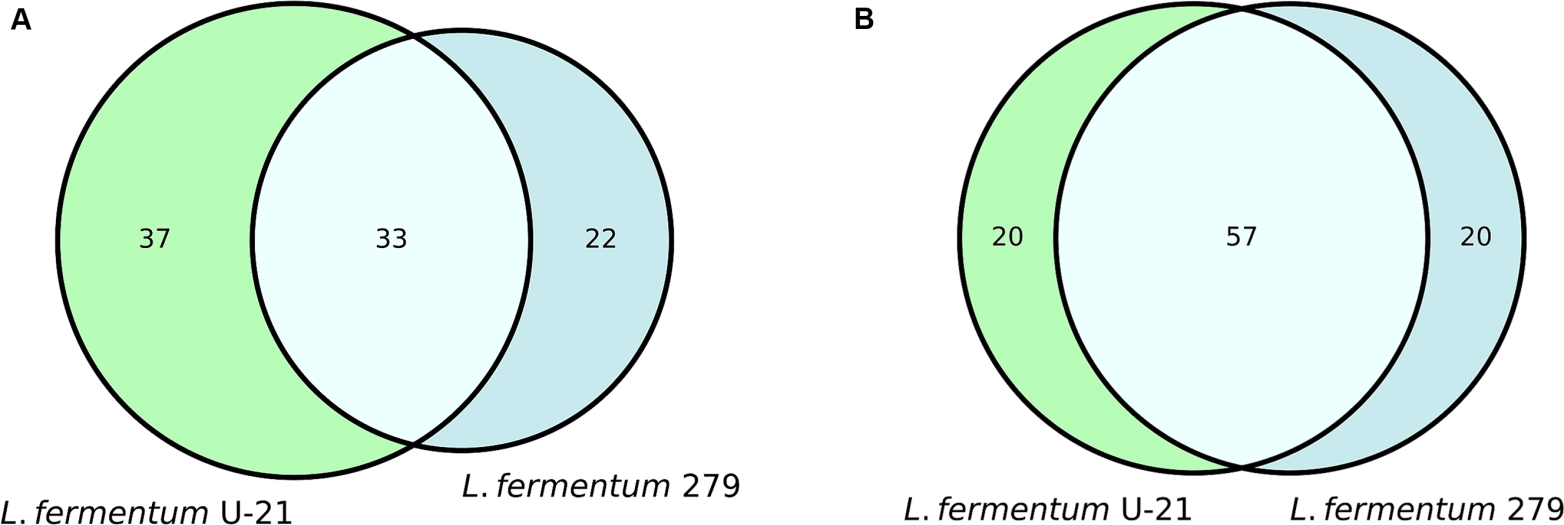
Common and unique metabolites in the Vienn diagram view: **(A)** in the vesicles of *L.fermentum* U-21 and *L.fermentum* 279; **(B)** in the cultural fluid, which include vesicles of *L.fermentum* U-21 and *L.fermentum* 279.

Quantitative metabolomic analysis of the *L.fermentum* U-21 strain revealed 77 metabolites in the culture fluid and 70 in the vesicles, 20 metabolites in the culture fluid and 37 in the vesicles are unique (Supplementary Tables S2, S3). *L.fermentum* U-21’s metabolites compared to the strain *L.fermentum* 279, which does not exhibit antioxidant (AO) properties (Figure 1).

We analyzed the 15 most represented metabolites in the culture fluid and looked at how they correlate with metabolites in the vesicles. 15 metabolites that are represented in the highest concentrations are shown in Table 2. The table shows that in the culture fluid and in vesicles the concentration of the corresponding metabolites increases in the same order.

**Table 2:** Top 15 most represented metabolites in culture fluid and vesicles of *L.fermentum* U-21.

	Cultural fluid	Vesicles
Metabolite	Concentration, μg/g	Concentration, μg/g
Lactic Acid	7,058	3.6
Propylene glycol	1,933	–
Pyroglutamic acid	348	0.85
D-Fructose	321	1.74
1-Hexadecanol	200	–
D-(-)-Tagatose	192	–
Butanedioic acid, (E)	169	1.94
L-Alanine	127	–
Glycerol	105	–
D-Glucose	104	1.98
D-Glucitol	94	–
L-Threonine	93	0.23*
DL-Phenylalanine	90	–
Aucubin	85	–
L-Leucine	83	0.04

Symbol * indicates concentrations of unique amino acid found in vesicles of *L.fermentum* U-21 strain compared to *L.fermentum* 279 strain.

It was detected 26 amino acids and peptides, 3 fatty acids and its glycerides, 32 polyols, 7 aromatic compounds and 7 hydroxy acids in the *L.fermentum* U-21’s cultural fluid (Supplementary Table S2). The identified standard amino acids can be divided into three groups depending on their concentration levels: the first group includes amino acids with concentrations ranging from 127 to 83 μg/g [L-Alanine (127 μg/g), L-Threonine (93 μg/g), L-Leucine (83 μg/g)], the second group ranges from 50 to 12 μg/g [L-Isoleucine (50 μg/g), L-Glutamic acid (46 μg/g), L-Valine (43 μg/g), L-Methionine (26 μg/g), L-Proline (21 μg/g), L-Aspartic acid (20 μg/g), L-Tyrosine (14 μg/g), L-Serine (12 μg/g)], and the third group ranges from 1.7 to 0.4 μg/g [L-Tryptophan (1.7 μg/g), Asparagine (0.5 μg/g) and Glycine (0.4 μg/g)]. Cysteine, arginine, histidine, glutamine and lysine were not detected.

There were 13 amino acids and peptides, 12 fatty acids and its glycerides, 20 polyols, 8 aromatic compounds and 7 hydroxy acids in the vesicles (Supplementary Table S3). The standard amino acids can be categorized into three groups based on their concentration levels. The first group consists of amino acids with concentrations ranging from 1.7 to 0.98 μg/g, and includes L-Glutamic acid (1.68 μg/g), L-Serine (1.06 μg/g), L-Aspartic acid (0.98 μg/g). The second group ranges from 0.25 to 0.1 μg/g [L-Methionine (0.25 μg/g), L-Threonine (0.23 μg/g), Glycine (0.11 μg/g)], while the third group ranges from 0.07 to 0.02 μg/g [L-Valine (0.07 μg/g), L-Leucine (0.04 μg/g) and L-Phenylalanine (0.02 μg/g)]. 6 of these amino acids such as L-Glutamic acid, L-Methionine, L-Threonine, Glycine, L-Valine, L-Phenylalanine are unique for *L.fermentum* U-21 in comparison with *L.fermentum* 279.

The proteoinogenic amino acids found in the culture fluid and vesicles of *L.fermentum* U-21 strain are presented in Table 3.

**Table 3:** Proteinogenic amino acids in culture fluid and vesicles of *L.fermentum* U-21.

	Cultural fluid	Vesicles
Amino acids	Concentration, μg/g	Concentration, μg/g
L-Alanine	127	–
L-Threonine	93	0.23*
L-Leucine	83	0.04
L-Isoleucine	50	–
L-Glutamic acid	46	1.68*
L-Valin	43	0.07*
L-Methionine	26	0.25*
L-Proline	21	–
L-Aspartic acid	20	0.98
L-Tyrosine	14	–
L-Serine	12	1.06
L-Tryptophan	1.7	–
Asparagine	0.5	–
Glycine	0.4	0.11*
L-Phenylalanine	-	0.02*

Symbol * indicates concentrations of unique amino acids found in vesicles of *L.fermentum* U-21 strain compared to *L.fermentum* 279 strain.

As a result of comparative analysis with *L.fermentum* 279 strain, 37 metabolites in the vesicles appeared to be unique for *L.fermentum* U-21 with the highest quantities being Octadecane (1.8 μg/g), L-Glutamic acid (1.7 μg/g), Tetracosane (1.5 μg/g), 2,3,4,5-Tetrahydroxypentanoic acid-1,4-lactone (1.3 μg/g), β-Hydroxypyruvic acid (1.1 μg/g), Erythro-pentonic acid, 2-deoxy (1 μg/g) (Supplementary Table S4).

Among 20 unique metabolites of *L.fermentum* U-21 strain’s culture fluid D-Galactose (17.1 μg/g), Arabinose (9.3 μg/g), 2,3,4,5-Tetrahydroxypentanoic acid-1,4-lactone (8 μg/g), 2-Deoxypentofuranose (6.8 μg/g), Scyllo-inositol (4.6 μg/g), Glyceric acid (4 μg/g), 9,12-Octadecadienoic acid (Z,Z) (3.4 μg/g), Myo-inositol phosphate (3 μg/g), Glycolic acid (1.9 μg/g) were represented in the highest concentration (Supplementary Table S5).

#### 3.1.1 Biologically active metabolites of *L.fermentum* U-21

According to the literature data, the biologically active and health beneficial metabolites, which are unique *L.fermentum* U-21 or for concentration of which are much more in the *L.fermentum* U-21’s cultural fluid and vesicles, were identified. Aucubin (85 μg/g), Scyllo-Inositol (4.6 μg/g), Cyclo(Leu-Gly) (0.35 μg/g) and Tryptophan (1.7 μg/g), Methionine (26 μg/g), Tyrosine (14 μg/g), which are amino acids with antioxidant potential, were found in the cultural fluid and Scyllo-Inositol (0.14 μg/g) and Niacin (0.018 μg/g) were found in vesicles. Methionine (0.25 μg/g) were also detected in the vesicles of *L.fermentum* U-21. *L.fermentum* U-21’s metabolites with health-beneficial properties are presented in Table 4.

**Table 4:** *L.fermentum* U-21’s metabolites with health-beneficial properties.

	Cultural fluid	Vesicles
Metabolite	Concentration, μg/g	Concentration, μg/g
Aucubin	85	–
Methionine	26	0.25*
Tyrosine	14	–
Scyllo-inositol	4.6*	0.14*
Tryptophan	1.7	–
Niacin	1.3	0.018
Cyclo(Leu-Gly)	0.35*	–
GABA	0.096	0.044

Symbol * indicates concentrations of unique metabolites found in cultural fluid and vesicles of *L.fermentum* U-21 strain compared to *L.fermentum* 279 strain.

Minimal concentration of compounds identified in cultural fluid and vesicles is 0.002 μg/g.

Additionally, we conducted a metabolomic analysis of the *Limosilactobacillus fermentum* U-21 fraction to identify and characterize minor but biologically significant components. During the analysis, traces of Niacin (0.01 μg/g) and gamma-aminobutyric acid (GABA) (0.096 μg/g) were detected at minimal concentrations, which may exert potential effects on the host microbiota and metabolic processes of the organism (Rashmi et al., 2018; Tuteja, 2019; Karunaratne et al., 2020).

#### 3.1.2 Aucubin: biosynthesis paths and genes of *L.fermentum* U-21

Aucubin was found in the culture fluid of the *L.fermentum* U-21 strain. Aucubin is an iridoid glycoside widely spread in the families *Cornaceae, Garryaceae, Orobanchaceae, Globulariaceae, Eucommiaceae, Scrophulariaceae, Plantaginaceae*, and *Rubiaceae* (Kartini et al., 2023). In accordance with its biosynthetic origin, the classical name iridoid refers to natural monoterpenoids, that is, secondary metabolites. Iridoids are often found in plants as glycosides and very rarely as non-glycosidic compounds. Aucubin is a glycoside whose aglycone (i.e., aucubigenin) binds to the glucose group using an O-glycosidic bond (National Center for Biotechnology Information, 2024). *In vitro* and *in vivo* studies indicate that aucubin has a wide range of activities, including anti-inflammatory, antioxidant, antidepressant, antidiabetic, antifibrotic, antimicrobial, anticancer, antihyperlipidemic, gastroprotective, cardioprotective, hepatoprotective, retinoprotective, neuroprotective, osteoprotective, and renoprotective (Zeng et al., 2020). There are studies that have shown that aucubin improves the symptoms or prognosis of Parkinson’s disease, Alzheimer’s disease, intracerebral hemorrhage, diabetic encephalopathy, epilepsy, anxiety and depression, and traumatic brain injury (Yang et al., 2022). Aucubin has also been confirmed to have a positive effect on intestinal flora and a therapeutic effect on intestinal problems caused by cancer (Shao et al., 2022). Secondary metabolites (aucubin and others) of health interest are known to influence the activation of the Nrf2 pathway to relieve inflammation and oxidative stress and their potential as a treatment for neurodegenerative diseases (Li et al., 2021; Moratilla-Rivera et al., 2023).

Aucubin biosynthesis begins with glycolysis products (acetyl CoA or pyruvate). There are two main ways of biosynthesis. The most common one begins with the conversion of acetyl CoA to mevalonate acid and then to geranyl pyrophosphate. The other way of synthesis is called MEP/DOXP pathway. It does not contain acetyl CoA and mevalonate acid. The described pathways may differ significantly from one organism to another and have different intermediate reaction products. The amino acid sequences of the enzymes that catalyze these reactions may also differ. For example, aucubin synthase is missing from databases due to the fact that it is very different in different organisms.

**Figure 2:**
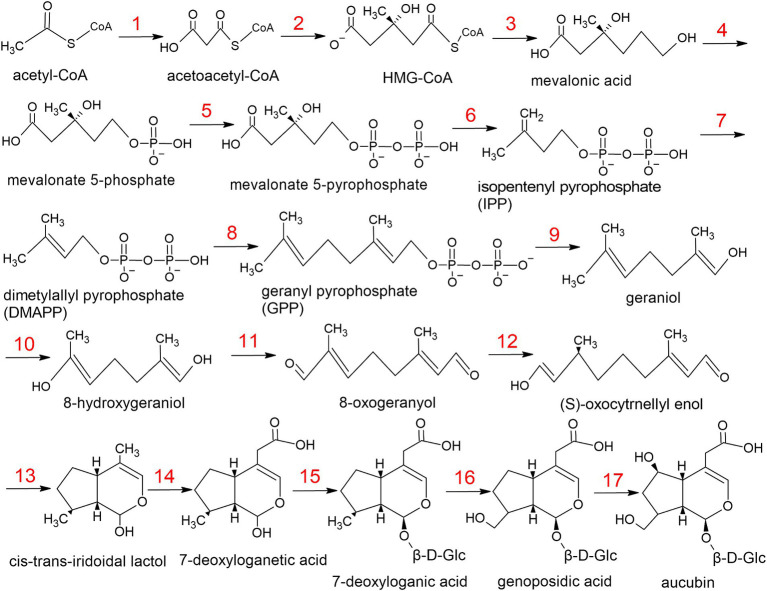
The metabolic pathway of aucubin in *L.fermentum* U-21.

For the *L.fermentum* strain U-21 aucubin synthesis is implemented through the mevalonate pathway (Figure 2, Table 5). The biosynthesis pathway was compiled based on information provided in database KEGG Pathway, as well as using scientific articles (McGarvey and Croteau, 1995; Sampaio-Santos et al., 2001; Christianson, 2017).

**Table 5:** Genes and enzymes of aucubin biosynthesis which were identified in strain *L.fermentum* U-21.

Step in pathway	Enzyme	Locus tag
1	acetyl-CoA acetyltransferase	C0965_RS05370
2	hydroxymethylglutaryl-CoA synthase	C0965_RS04175
3	hydroxymethylglutaryl-CoA reductase	C0965_RS01175
4	mevalonate kinase	C0965_RS06855
5	phosphomevalonate kinase	C0965_RS06865
6	mevalonate pyrophosphate decarboxylase	C0965_RS06860
7	isopentenyl pyrophosphate delta-isomerase	C0965_RS06870
8	geranyltranstransferase	C0965_RS07160
9	hydrolyze geranyl pyrophosphate	C0965_RS00160
10	geraniol 8-hydroxylase	C0965_RS03105
11	8-hydroxygeraniol oxidoreductase	C0965_RS01115
12	(S)-8-oxocytronellyl enol synthase	C0965_RS02015
13	iridoid synthase	C0965_RS05065
14	iridoid oxidase	C0965_RS03910
15	7-deoxyloganetic acid glucosyl transferase	C0965_RS03465
16	7-deoxyloganic acid hydroxylase	C0965_RS04785
17	aucubin synthase	Not included in any databases

The detected genes do not form an operon. They are distributed throughout the genome of *L.fermentum* U-21. Homologous proteins can be found in the Supplementary Table S6.

Genes are represented as locus tags. The number of the steps in the pathway matches the corresponding number in Figure 2.

#### 3.1.3 Niacin: biosynthesis paths and genes of *L.fermentum* U-21

Niacin was found both in vesicles and in the culture fluid of the *L.fermentum* U-21 strain. Regarding niacin biosynthesis (Table 6), microorganisms synthesize quinolinate, which, apparently, is a precursor for the synthesis of niacin in all living forms, from aspartate and dihydroxyacetone phosphate (Henderson, 1983). Niacin contains two types of vitamins, nicotinic acid and nicotinamide, which create the coenzymatic forms NAD and NADP. These coenzymes are essential for oxidative reactions that produce energy, but they also serve as substrates for enzymes involved in non-redox signaling pathways. This allows them to regulate biological functions such as cell cycle progression, cell death, gene expression, DNA repair. Niacin has been widely recognized as a crucial mediator of neuronal development and survival in the central nervous system (Gasperi et al., 2019). Nicotinamide speeds up the process of embryonic stem cells or neural progenitors becoming postmitotic neurons, which impacts neurogenesis (Griffin et al., 2013, 2017). Additionally, nicotinamide supports neuronal survival, particularly in oxidative stress situations, through various mechanisms (Chong et al., 2004). The Chicago Health and Aging Project (CHAP) study proposes that dietary niacin may guard against Alzheimer’s disease and age-related cognitive decline (Morris et al., 2004). Moreover, appropriate levels of niacin are necessary to decrease oxidative stress and neuroinflammation, which is involved in Parkinson’s disease development (Wakade et al., 2018).

**Table 6:** Genes and enzymes of niacin biosynthesis which were identified in strain *L.fermentum* U-21.

Reaction in the pathway	Enzyme	Locus tag
beta-nicotinate D-ribonucleotide → deamido-NAD^+^	nicotinate-nucleotide adenylyltransferase	C0965_RS07390
deamino-NAD^+^ < => NAD^+^	ammonia-dependent NAD(+) synthetase	C0965_RS01640
NADPH + NAD^+^ → NADP^+^ + NADH	NAD(P) + transhydrogenase	C0965_RS06595
NAD^+^ → NADP^+^	NAD+ kinase	C0965_RS03395
a 5′-ribonucleotide → a ribonucleoside	5′-ribonucleotide phosphohydrolase HAD (haloacid dehalogenase)-IIA family hydrolase YutD family protein	C0965_RS03240
		C0965_RS03235
5′-deoxyadenosine → adenine	5′-methylthioadenosine/ adenosylhomocysteine nucleosidase	C0965_RS03525
beta-nicotinate D-ribonucleotide → pyridine-2,3-dicarboxylate	nicotinate phosphoribosyltransferase	C0965_RS01635
		C0965_RS07910
beta-nicotinate D-ribonucleotide → nicotinate	Nicotinate phosphoribosyltransferase	C0965_RS04960
NAD(H) → NMN(H)	NAD+ diphosphatase	C0965_RS09705

Homologous proteins can be found in the Supplementary Table S7.

Genes are represented as locus tags.

### 3.2 Proteomic analysis of the *L.fermentum* U-21 and *L.fermentum* 279 strains

#### 3.2.1 Comparative electrophoretic analysis of proteins in different fractions from *L.fermentum* U-21 and *L.fermentum* 279 strains

**Figure 3:**
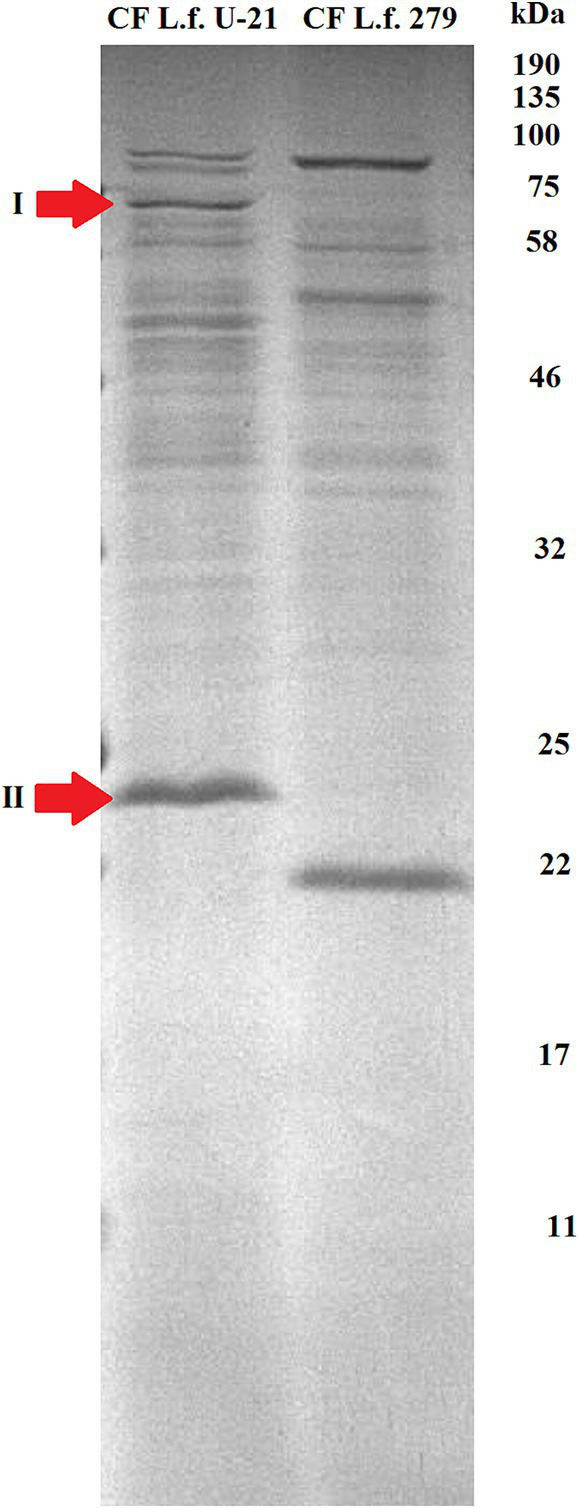
Electrophoretic separation of proteins in the fraction of cultural fluid of the *L.fermentum* U-21 (L.f. U-21) and the *L.fermentum* 279 (L.f. 279) strains. Notable bands of proteins are selected and marked as possible biomarkers: I—ATP-dependent Clp protease ATP-binding subunit (ClpL); II—LysM domain containing protein. *CF*—cultural fluid.

**Figure 4:**
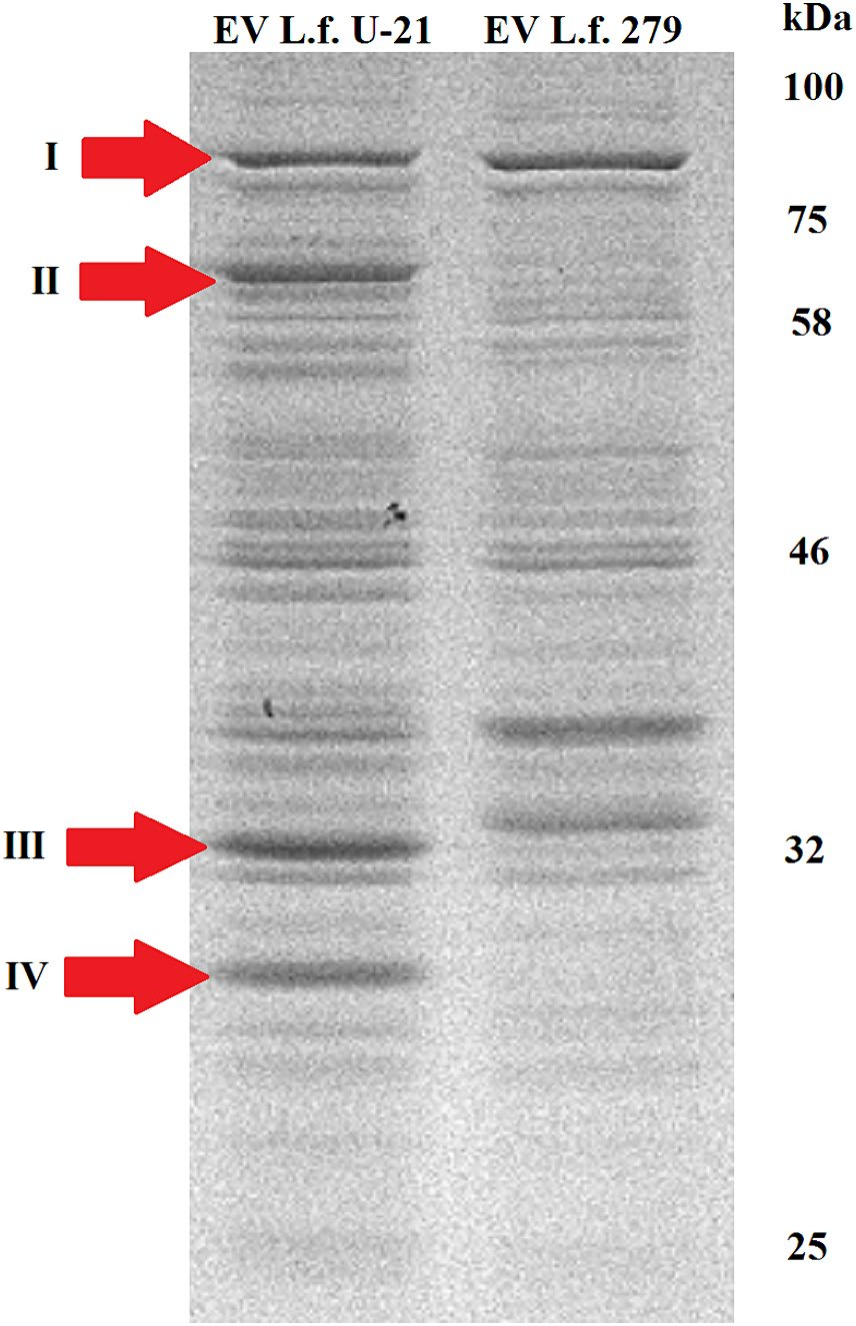
Electrophoretic separation of proteins in the fraction of extracellular vesicles of the *L.fermentum* U-21 (L.f. U-21) and *L.fermentum* 279 (L.f. 279) strains. The following proteins in the vesicles of *L.fermentum* U-21 were identified as possible biomarkers: I—phosphoketolase, II—ATP-dependent Clp protease ATP-binding subunit (ClpL), III—30S ribosomal protein S2, IV—NlpC/P60 domain containing protein.

Proteins from the bacterial cell (TE—total extract), cultural fluid (*CF*) and extracellular vesicles (EV) of the *L.fermentum* U-21 and the *L.fermentum* 279 strains were separated by SDS PAGE. Using mass spectrometric analysis, some proteins presented in the extracellular vesicles of the *L.fermentum* U-21 strain were identified as possible biomarkers (Figures 3, 4). The cells were grown to a stationary phase before conducting the electrophoresis.

Although electropherograms of TE samples of *L.fermentum* U-21 and *L.fermentum* 279 strains looked similar we can see differences in protein fractions of *CF* and EVs between the studied *L.fermentum* U-21 and comparative *L.fermentum* 279 strains. There are visible differences in the fraction of cultural fluid with a range of molecular weights about 75–100 kDa. We can also see a different light-weighted protein in this fraction of both strains with molecular weights about 25 and 22 kDa, respectively.

There are visible differences in protein composition of EV fraction as well. The identified ATP binding subunit of Clp protease (ClpL) (AI Ebrahim et al., 2024) with a molecular weight of 76.7 kDa (II) is more abundant in the vesicles of *L.fermentum* U-21 strain than in the vesicles of comparative strain. Also there is a notable band identified as NlpC/P60 domain containing protein (IV) in the vesicles of *L.fermentum* U-21.

#### 3.2.2 Proteomic mass spectrometric analysis of the extracellular vesicles fraction of *L.fermentum* U-21

A mass spectrometric analysis was conducted on the Extracellular vesicles’ protein fraction of the *L.fermentum* U-21 strain. The total amount of detected proteins is 758. For further analysis of the output table of MaxQuant protein groups, which are known “contaminants,” “only identified by site” or “reverse,” were removed. Additionally, proteins were considered identified reliably if at least two peptides were found for them. After filtering, the number of identified proteins decreased to 453. Top of 30 most-abundant proteins according to MS-data may be found in Supplementary Table S8.

List of proteins was analyzed by the bioinformatic resource DAVID.^3^ Some proteins were grouped according to their molecular function (GOTERM MF) and biological process (GOTERM BP) using. However, DAVID could identify less than a half of protein entries by which 250 proteins had been left unrecognized.

Table with mass spectrometry data for proteomic analysis of EVs of *L.fermentum* U-21 is available via ProteomeXchange with identifier PXD050857.

**Figure 5:**
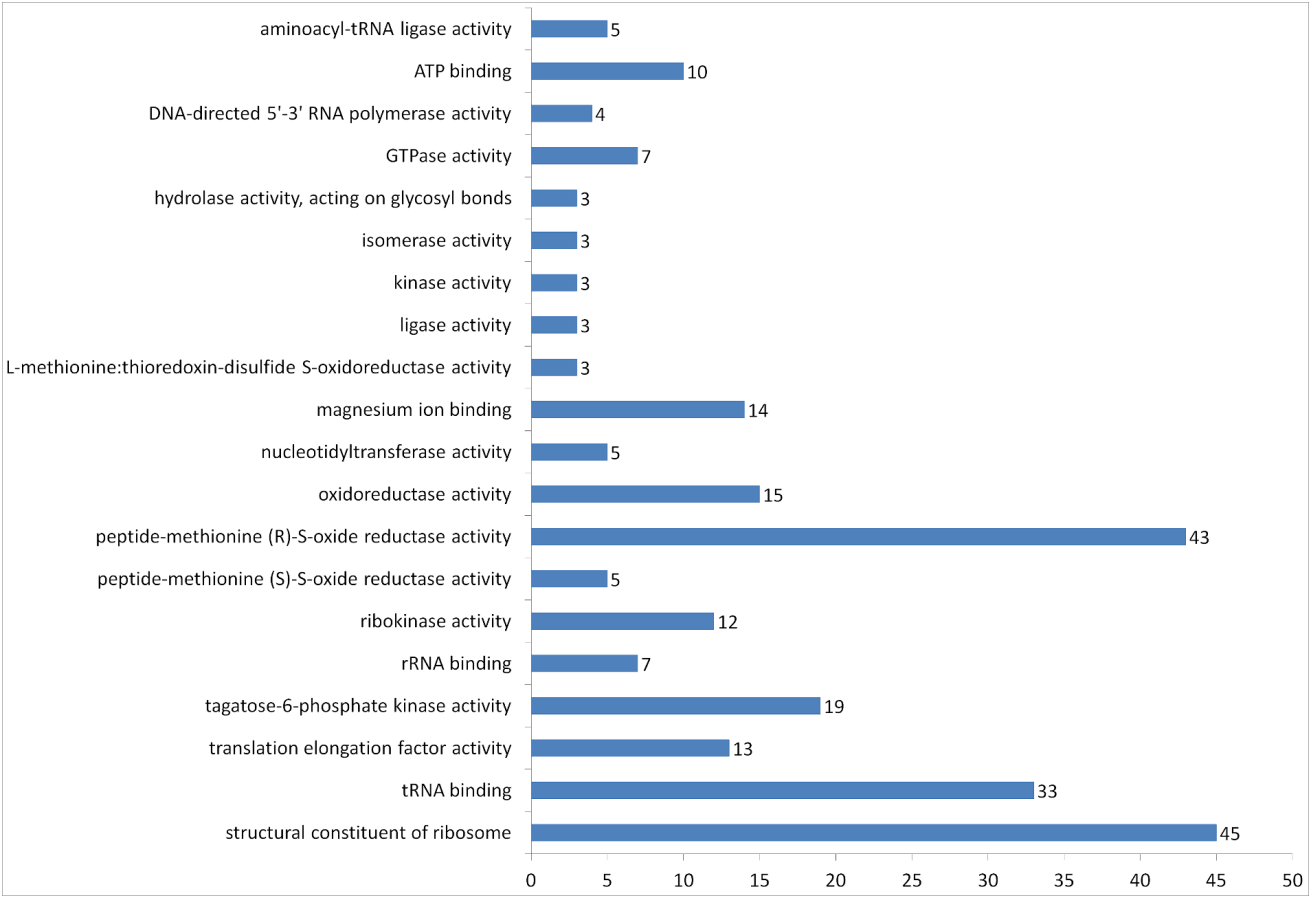
Number of proteins in vesicles of *L.fermentum* U-21 grouped according to their GOTERM molecular function by DAVID.

According to the results of DAVID’s protein separation on molecular functions, 250 entries of proteins were grouped into 20 groups (Figure 5). The most abundant groups were structural constituent of ribosome, peptide-methionine (R)-S-oxide reductase activity and tRNA binding. We can also see a group of entries with oxidoreductase activity, which may include proteins with antioxidant properties.

**Figure 6:**
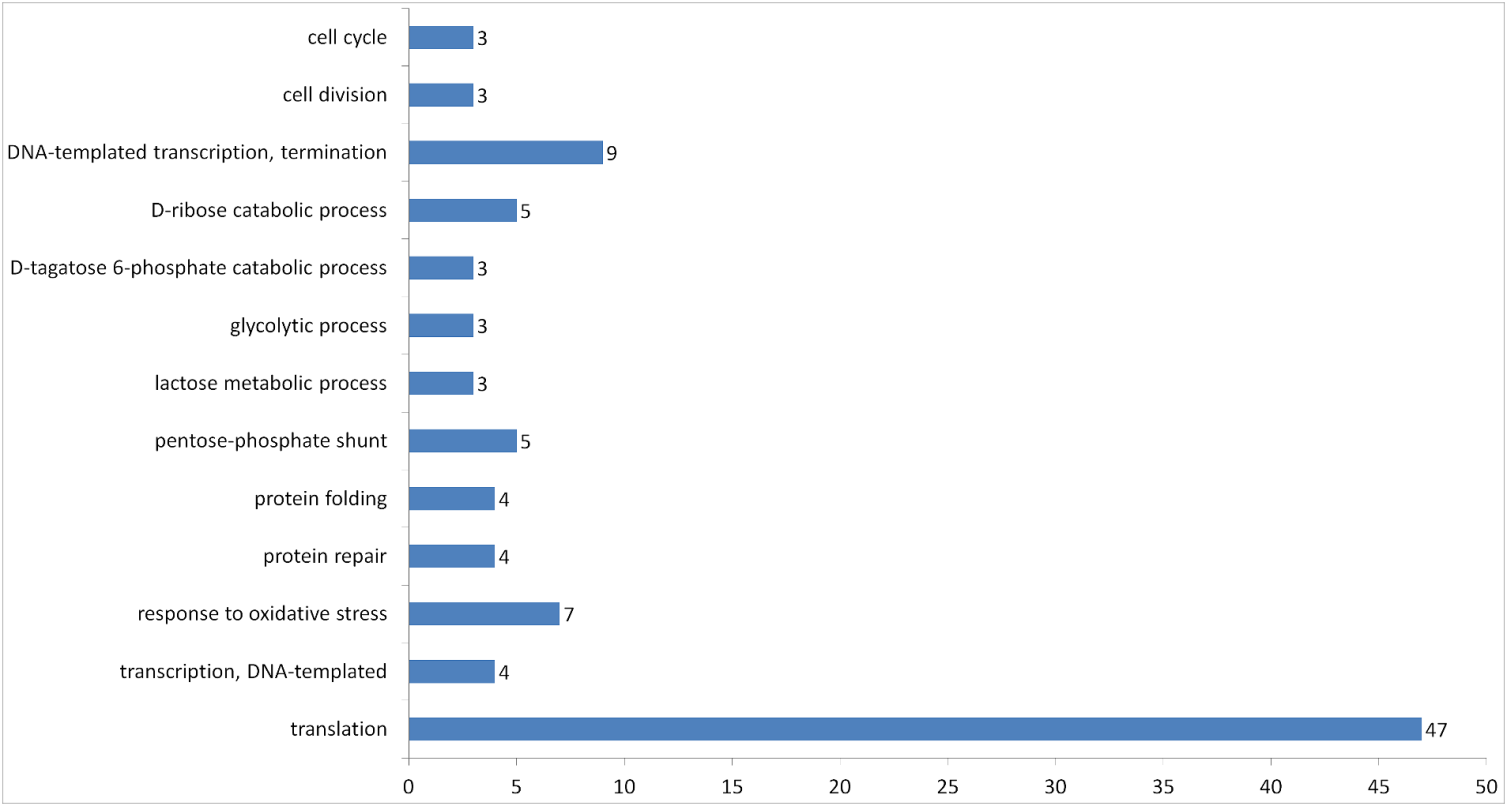
Number of proteins in vesicles of *L.fermentum* U-21 grouped according to their GOTERM biological process by DAVID.

Additionally, 100 entries of proteins were grouped into 13 biological processes by DAVID (Figure 6). The most abundant group was related to the translational process, which includes the majority of ribosomal proteins found in the vesicles.

In accordance with earlier published data proteins related to the anti-inflammatory activity were selected in Table 7 (Averina et al., 2021).

**Table 7:** Proteins of *L.fermentum* U-21 with a potential anti-inflammatory activity.

Protein groups	Proteins of *L.fermentum* U-21
Antioxidant proteins	Thioredoxin reductase, NAD(P)/FAD-dependent oxidoreductase, Thiol peroxidase, MFS transporter, DNA-protecting protein DprA
Immunomodulatory proteins	LysM peptidoglycan-binding domain-containing protein, NlpC/P60 domain-containing protein
Chaperone proteins	ATP-dependent Clp protease ClpL, Chaperonin GroEL, Co-chaperonin GroES, Chaperone protein DnaK, Protein GrpE, 33 kDa chaperone hslO,

### 3.3 Assessment of *L.fermentum* U-21 strain’s immunomodulatory effects

**Figure 7:**
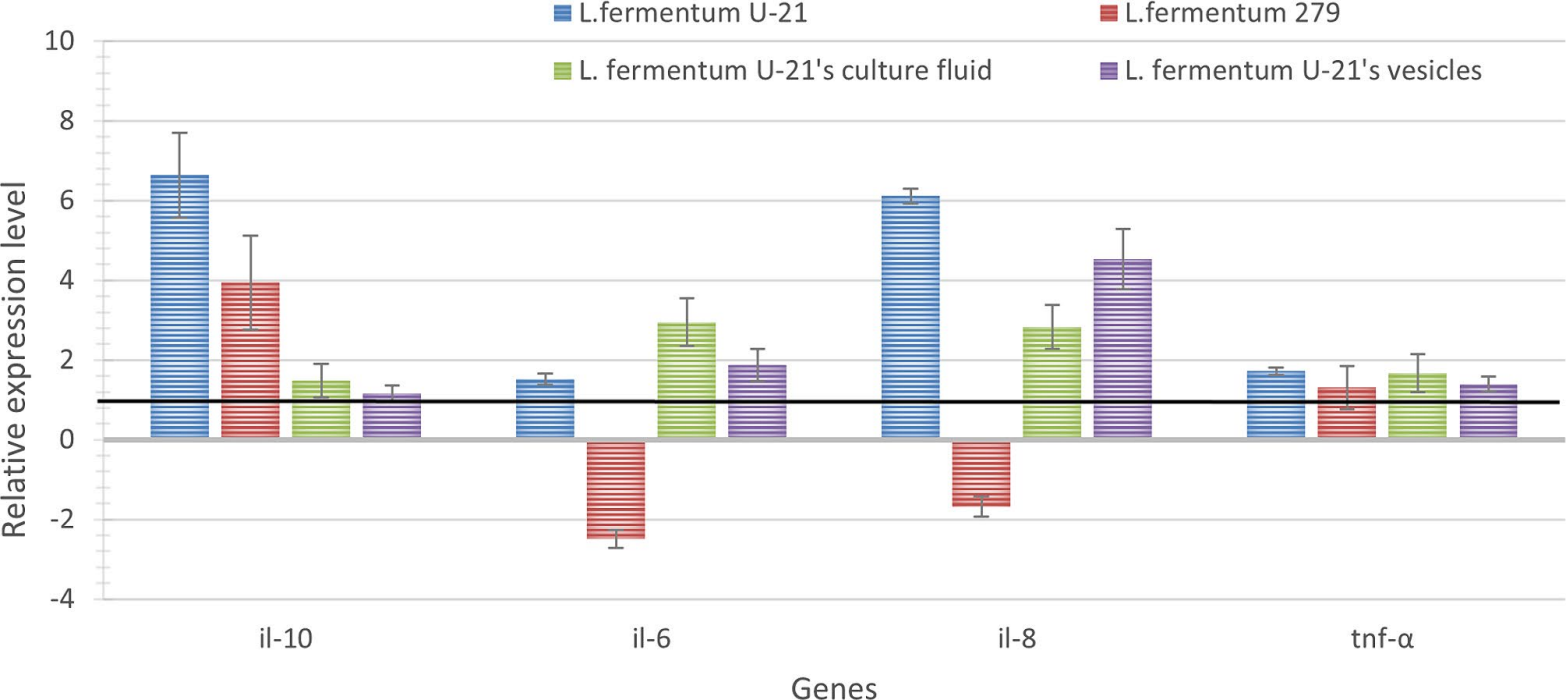
The relative level of expression of pro-inflammatory *il-6, il-8, thf-α* and anti-inflammatory *il-10* cytokine genes in the studied samples after 3 h of coincubation with live bacteria, culture fluid and vesicles. Expression of the studied genes of the LPS-induced THP-1 cells without adding any bacterial samples was considered equal to 1 (black line—control for samples which was incubated with live bacterial cells and vesicles). Expression of the studied genes of the LPS-induced THP-1 cells with the addition of MRS medium was considered equal to 1 (black line—used as a control for samples of THP-1 cells incubated with the culture liquid of the *L.fermentum* U-21 strain). The error bars represent the standard deviation calculated from three independent replicates.

To determine the immunomodulatory activity of *L.fermentum* U-21 strain, its cultural fluid and vesicles, THP-1 cells were treated with LPS (1 μg/mL; 3 h) and with bacterial samples (live bacterial cells, vesicles, and culture liquid) and the gene expression of pro-inflammatory cytokines IL-6, IL-8, TNF-α and anti-inflammatory cytokine IL-10 were measured after 3 h. The relative expression levels of the studied genes are shown in Figure 7.

As a result of the analysis, *L.fermentum* U-21 strain’s live bacteria significantly in-crease the expression of pro-inflammatory cytokines IL-8 and anti-inflammatory IL-10 by 6.1 and 6.6 times, respectively, and pro-inflammatory cytokines IL-6, TNF-α in-creases slightly. On the other hand, live bacteria of the *L.fermentum* 279 strain have little effect compared to the effect of the *L.fermentum* U-21 strain on changes in the gene expression of all studied cytokines: it increases the expression of IL-10 by 4 times and reduces IL-6 by 2.5 times.

It was shown that the culture fluid of *L.fermentum* U-21 has a more pronounced immunomodulatory effect, increasing the expression of cytokines IL-6 and IL-8 by 2.9 and 2.8 times. Moreover, it has been demonstrated that the vesicles significantly affect the expression of the cytokine IL-8 and IL-6, increasing the expression by 4.5 and 1.9 times; the expression of other cytokines changes slightly.

## 4 Discussion

In recent years, there has been an increasing tendency to create drugs of various types based on certain commensal bacteria of the human microbiota and their ingredients. This primarily concerns live biotherapeutic products (LBPs) (Ağagündüz et al., 2022) and postbiotics (Ma et al., 2023). The creation of such drugs, which can be called pharmacobiotics, requires an understanding of the mechanisms of their action and the identification of pharmacologically active ingredients that determine their target properties. As a rule, this is a complex of biologically active substances synthesized by a specific strain, promoted as LBP or postbiotics (including vesicles): proteins, enzymes, low molecular weight metabolites, small RNAs, etc. (Danilenko et al., 2021; Yunes et al., 2022).

The *L.fermentum* U-21 strain studied in this article is a promising candidate for the creation of an LBP and a postbiotic drug for the complex treatment of neurodegenerative diseases, in particular Parkinsonism. The strain underwent a series of studies *in vitro* and *in vivo*, which showed the effectiveness of its action on models of Parkinsonism: “*E.coli* – paraquat,” “*C.elegans* – paraquat,” “*Rodents* – paraquat” (Marsova et al., 2020). The safety of its use has been established in several biological tests. This gave rise to a body of research to identify and characterize low molecular weight substances, proteins and enzymes that could potentially be responsible for its anti-inflammatory properties (including neuroinflammation), as well as the refolding of misassembled proteins.

Several mechanisms of protective and anti-inflammatory action caused by *L.fermentum* U-21 can be assumed. Neutralization of radicals and reactive oxygen species by proteins of the thioredoxin complex. Restoration of the intestinal barrier, modulation of the composition of the intestinal microbiota, leading to a decrease in systemic inflammation. Refolding of degraded proteins formed as a result of oxidative stress, including in neurons of the enteric and central nervous systems.

In this work, we used proteomic and metabolomic analysis to identify proteins, enzymes, and low molecular weight compounds in cells, culture medium, and vesicles of the *L.fermentum* U-21 strain. As an object of comparison, we used the strain *L.fermentum* 279, which did not exhibit noticeable antioxidant properties in previous studies (Marsova et al., 2018).

Table 8 summarizes the results of the use of omics technologies and genomic analysis of *L.fermentum* U-21. The table presents 4 groups of substances: antioxidants, immunomodulators, neuromodulators and disaggregases. In each group, we selected substances that, in our opinion, are most likely responsible for the properties of *L.fermentum* U-21 in the context of innovative trends in the development of drugs aimed at treating neurological diseases (Parkinsonism, Alzheimer’s, etc.). These are neuroanti-inflammatory drugs, immunomodulators selective for the functioning of the nervous system and substances that disaggregate improperly assembled neuronal proteins. Potential disaggregase substances found in *L.fermentum* U-21 can be divided into three groups: chaperone proteins (ClpL, DnaK, DnaJ, GrpE), chemical chaperones (scyllo-inositol) and pharmacological chaperone (tryptophan) (Katikaridis et al., 2021; Almeida and Brito, 2022; Khan and Khan, 2022; Wen et al., 2023).

**Table 8:** Key compounds of *L.fermentum* U-21 strain with disaggregase, antioxidant, immunomodulatory and neuromodulatory activity, which were identified as a result of proteome and metabolome analysis.

Compound group	*L.fermentum* U-21		
	Proteins	Metabolites	Genes responsible for synthesis
Disaggregases	*ATP-dependent Clp protease ClpL, *Chaperone protein DnaK, *Protein GrpE	*Scyllo-Inositol, Tryptophan	ClpL: C0965_RS00195 DnaK: dnaK GrpE: grpE
Antioxidant	*Thioredoxin reductase, *NAD(P)/FAD-dependent oxidoreductase, *Thiol peroxidase	Aucubin	Aucubin: mvk, fni, der, murG, mmuM TR: trxB NAD(P)/FAD-DO: C0965_RS06310 TP: tpx
Immunomodulatory	*LysM peptidoglycan-binding domain-containing protein, *NlpC/P60 domain-containing protein	*Niacin	Niacin: nadD, nadE, pntA, ppnK, pynA, pncB LysM: C0965_RS02490 NlpC/P60: C0965_RS08015
Neuromodulatory	—	*Niacin *GABA cyclo(Leu-Gly) Aucubin	—

Proteins and metabolites found in extracellular vesicles of *L.fermentum* U-21 are marked with an asterisk (*).

Thioredoxins are small proteins with a molecular weight about 12 kDa. They form and break disulfide bonds by adapting redox potential in the cell. The family of pyridine nucleotide-disulfide oxidoreductase also includes glutathione reductase (GR), trypanothione reductase (TryR), alkyl hydroperoxide reductase, lipoamide dehydrogenase, and mercuric reductase (Lu and Holmgren, 2014).

A protein containing a NlpC/P60 domain serves mostly for peptidoglycan remodeling. NlpC/P60 are usually about 150 AA in length and have a molecular weight about 15 kDa. They act as endopeptidases on the peptide chain in peptidoglycan. After being released these small muramyl oligopeptides often act as signal molecules activating non-specific immune responses in the human gut (Griffin et al., 2023). A NlpC/P60 domain may be a part of a protein which contains a LysM peptidoglycan binding domain as well. The main function of the LysM domain is to bind peptidoglycan parts (Buist et al., 2008).

Scyllo-Inositol found in the culture fluid and in the vesicles of the strain is a chemical chaperone. It is a unique metabolite for *L.fermentum* U-21 strain compared to *L.fermentum* 279. Chemical chaperones are small compounds that have the ability to either maintain the natural structure of polypeptide chains or disrupt and disassemble misfolded or clumped states (Almeida and Brito, 2022). Therefore, Scyllo-Inositol can be used in the treatment of amyloid diseases. In literature, it has been demonstrated that Scyllo-Inositol can hinder or decrease the formation of abnormal huntingtin clumps in Huntington’s disease (Lai et al., 2014) and α-synuclein plaques in Parkinson’s disease (Ibrahim and McLaurin, 2016). Furthermore, administering Scyllo-Inositol has been found to decrease cognitive impairment synaptic toxicity and lower amyloid-β fibrils and plaques in a mouse model (McLaurin et al., 2006), as well as in clinical trials for Alzheimer’s disease (Salloway et al., 2011).

In addition to Scyllo-Inositol, the amino acid Tryptophan was found in the culture fluid of the *L.fermentum* U-21 strain. Tryptophan may be considered a pharmacological chaperone and also be used in the fight against the amyloid diseases. Pharmacological chaperones are appealing for potential therapeutic use due to the ability to exert effects similar to molecular chaperones while requiring lower concentrations than chemical chaperones (Almeida and Brito, 2022). *In vitro* and *in vivo* experiments have demonstrated that the combination of naphthoquinones and tryptophan has an active role in preventing aggregation in diverse amyloid systems, including amyloid-β, islet amyloid polypeptide, tau, and α-synuclein (Scherzer-Attali et al., 2012; Krishna Kumar et al., 2018; Viswanathan et al., 2019; Paul et al., 2019a,b).

Our research demonstrates for the first time the ability of *L.fermentum* bacteria to synthesize aucubin, a substance usually secreted by plants: genes potentially responsible for its synthesis have been identified. Aucubin has a wide range of effects, where the major one is antioxidant properties. It can help neutralize harmful free radicals in the body (Yang et al., 2022; Kartini et al., 2023). This property may contribute to its protective effects against oxidative stress and its associated health issues. When using aucubin arises the problem of its insolubility and low bioavailability (Zeng et al., 2020). These problems are an obstacle to the use of aucubin for therapeutic purposes. The ability of the commensal bacterium of the human intestinal microbiota *L.fermentum* U-21 to synthesize aucubin may help solve the problem of bioavailability in target tissues and human organs.

Nicotinic acid (niacin) was found in the vesicles and culture fluid of the *L.fermentum U-21.* It is essential for the optimal functioning of the nervous system, and DNA repair. Niacin regulates the production of inflammatory molecules called cytokines (Montserrat-de la Paz et al., 2017). By modulating cytokine production, nicotinic acid helps to maintain immune homeostasis and prevent excessive inflammation. Research has shown that nicotinic acid can influence the activity of certain immune cells, such as macrophages and lymphocytes, by enhancing their function and promoting a balanced immune response. Niacin has a vital role in the synthesis of neurotransmitters, such as serotonin, dopamine, and norepinephrine, which are essential for mood regulation, cognition, and overall brain function (Nogueira-de-Almeida et al., 2023). In this way it can modulate the function of the nervous system. These properties of niacin are crucial in the development of treatment methods for neurodegenerative diseases.

γ-aminobutyric acid (GABA) was found in the vesicles and culture fluid of the *L.fermentum* U-21 strain. GABA is the major neurotransmitter in the mammalian nervous system that enables rapid inhibitory synaptic transmission. GABA also stabilizes neuronal activity, whereas an imbalance between excitatory and inhibitory neurotransmitters can lead to neurodegenerative diseases (Rashmi et al., 2018). Additionally, modern research indicates that GABA may have antidepressant activity, hepato-protective, reno-protective and intestinal-protective properties (Yunes et al., 2016, 2020; Averina and Danilenko, 2017; Ngo and Vo, 2019).

Vesicles are membrane bubbles that can contain various substances such as proteins, lipids, carbohydrates, DNA, mRNA. Bacteria use vesicles to transmit signals among themselves and to interact with the environment. Microbiota bacteria living in the gut produce various neurotransmitters, metabolites that can be packaged into vesicles and then transported through the blood or lymphatic system, crossing tissue barriers, including the blood–brain barrier, to various organs as well as the brain (Haas-Neill and Forsythe, 2020; Cuesta et al., 2021). One study showed that extracellular vesicles can enhance antioxidant activity by improving the physical intestinal barrier and remodeling the gut microbiota. Unlike probiotics, which must be pre-colonized, vesicles can act directly on the certain organ (Feng et al., 2023). All this indicates the possibility of using bacterial vesicles as carriers for the delivery of biologically active molecules, their combinations to various tissues and organs of the host organism to correct their normal functioning. Vesicles of the *L.fermentum* U-21 strain contain a number of proteins and low molecular weight metabolites with pharmacologically active properties. Learning to control their composition in vesicles is the most important task.

However, this area of research is relatively new and actively developing, and additional research is needed to more fully understand the mechanisms of substance delivery to the brain. It is important to note that each bacterium can secrete different components packing them into vesicles and affect the brain in different ways.

LBPs, culture fluid-based drugs (postbiotics), and extracellular vesicles exhibit different immunomodulatory activities in a cellular model, reflecting their structural and functional differences and the composition of bioactive ingredients. This suggests that they can be used to create drugs and functional food products for various purposes.

## 5 Conclusion

With the expansion of our knowledge about the role of the intestinal microbiota in the development of diseases of various etiologies, including cancer, autoimmune and neurodegenerative, there comes a clear understanding of the need and possibility of correcting the microbiome to achieve homeostasis of a healthy body. Most diseases are accompanied by inflammatory processes in various organs and tissues, leading to dysfunction of the endocrine, immune and nervous systems. Existing targeted monotherapy drugs no longer provide the desired effect in treatment for various reasons. The use of herbal multicomponent products is also not always effective due to their poor bioavailability.

Probiotic bacteria, widely used as dietary supplements, are intended for healthy people and do not have proven medical properties for use in medical practice. LBP is a category of drugs that is based on the selection of unique strains of human commensal bacteria with specified properties for the treatment of a specific nosology. Today, the entire scientific community understands the target strain-specific selection of commensal bacteria for the creation of LBP. Comparative genomics and omics technologies are the tools needed to select and characterize a target strain for LBP generation. Sub-sequent preclinical and clinical studies will confirm its suitability for medical practice.

The development of metagenomic and omics technologies, the accumulated volume and quality of knowledge about the gut microbiome allows us to consider it as a source for the creation of nature-like drugs for various purposes. LBP preparations and postbiotics are promising for the creation of drugs for the treatment and prevention of neurological diseases, including Parkinsonism. *L.fermentum* U-21 strain showed unique antioxidant, antineuroinflammatory and immunomodulatory properties in *in vitro* and *in vivo* experiments. In the present work we identified proteins (chaperones, enzymes of antioxidant complex, etc.) and low molecular weight metabolites (aucubin, niacin, GABA) potentially determining the properties of *L.fermentum* U-21 strain and postbiotics based on it, including vesicles. Thus, the integrated use of omics technologies for the example of *L.fermentum* U-21 allowed us to develop approaches for the promotion of other probiotic strains as LBP and postbiotic products including vesicles. The use of extracellular vesicles of bacteria from the human intestinal microbiota as natural means of delivering pharmacologically active ingredients to the desired human organ looks extremely promising. It seems promising to carry out target screening of existing Research Topics of *Lactobacillus* and *Bifidobacterium* strains for the presence of genes, proteins and metabolites with specified properties. The strains selected in this way should be tested on adequate rodent models.

## Data availability statement

The HPLC-MS/MS data have been deposited to the ProteomeXchange Consortium via the PRIDE (Perez-Riverol et al., 2022) partner repository with the dataset identifier PXD050857.

## Ethics statement

Ethical approval was not required for the studies involving humans because we did not use animals our research. We used only standart test-system on THP-1 cell line. The studies were conducted in accordance with the local legislation and institutional requirements. The human samples used in this study were acquired from gifted from another research group. Written informed consent to participate in this study was not required from the participants or the participants’ legal guardians/next of kin in accordance with the national legislation and the institutional requirements.

## Author contributions

MO: Conceptualization, Investigation, Methodology, Writing – original draft, Writing – review & editing. DM: Conceptualization, Investigation, Methodology, Visualization, Writing – original draft, Writing – review & editing. AN: Conceptualization, Formal analysis, Investigation, Writing – original draft, Writing – review & editing. OT: Data curation, Methodology, Writing – review & editing. NAS: Data curation, Methodology, Writing – review & editing. DR: Conceptualization, Formal analysis, Investigation, Visualization, Writing – original draft, Writing – review & editing. OG: Data curation, Formal analysis, Visualization, Writing – original draft, Writing – review & editing. AAV: Methodology, Writing – original draft, Writing – review & editing. NMS: Investigation, Methodology, Writing – review & editing. ARV: Investigation, Writing – review & editing. SP: Resources, Writing – review & editing. VD: Conceptualization, Funding acquisition, Project administration, Resources, Supervision, Writing – original draft, Writing – review & editing.

## Footnotes

^1^https://www.kegg.jp/pathway/lfe00900

^2^https://www.kegg.jp/entry/map00760

^3^https://david.ncifcrf.gov
