# Supplementary material for: The use of omics technologies in creating LBP and postbiotics based on the Limosilactobacillus fermentum U-21
Source: Front Microbiol. 2024 Jun 11;15:1416688. doi: 10.3389/fmicb.2024.1416688 (PMC11197932; doi:10.3389/fmicb.2024.1416688)
Supplement: Supplementary file 1 [file Table_1.DOCX]

Supplementary Material

**Supplementary Table 1.** List of primers used in the study.

| **Primer name** | **Protein name** | **5’-> 3’ sequence** |
| --- | --- | --- |
| *gapdh-f* | GAPDH | GGAAGGTGAAGGTCGGAGTC |
| *gapdh-r* |  | TCAGCCTTGACGGTGCCATG |
| *il-6-f* | IL-6 | AAATTCGGTACATCCTCGAC |
| *il-6-r* |  | CAGGAACTGGATCAGGACTT |
| *il-8-f* | IL-8 | TGGCTCTCTTGGCAGCCTTC |
| *il-8-r* |  | TGCACCCAGTTTTCCTTGGG |
| *il-10-f* | IL-10 | TCAGGGTGGCGACTCTAT |
| *il-10-r* |  | TGGGCTTCTTCTAAATCGTTC |
| *tnf-α-f* | TNF-α | TCTCGAACCCCGAGTGACAA |
| *tnf-α-r* |  | TATCTCTCAGCTCCACGCCA |

**Supplementary Table 2.** Metabolites of *L.fermentum* U-21’s culture fluid divided by main classes

| **Metabolite name^1^** | **Concentration, μg/g** | **Class** |
| --- | --- | --- |
| Pyroglutamic acid | 347.808 | Amino acids and peptides |
| L-Alanine | 126.728 |  |
| L-Threonine | 93.427 |  |
| DL-Phenylalanine | 89.82 |  |
| L-Leucine | 82.974 |  |
| L-5-Oxoproline | 62.161 |  |
| L-Isoleucine | 49.859 |  |
| L-Glutamic acid | 45.881 |  |
| L-Valine | 43.106 |  |
| L-Methionine | 26.271 |  |
| L-Proline | 20.628 |  |
| L-Aspartic acid | 19.888 |  |
| L-Tyrosine | 14.245 |  |
| L-Serine | 12.025 |  |
| Cyclo(Phe-Pro) | 8.973 |  |
| Cyclo(Met-Met) | 5.458 |  |
| Cyclo(Pro-Val) | 3.885 |  |
| Cyclo(Ala-Pro) | 3.515 |  |
| L-Tryptophan | 1.665 |  |
| N-Formylglycine | 1.665 |  |
| Cyclo(Pro-Cly) | 1.573 |  |
| Asparagine | 0.544 |  |
| Glycine | 0.389 |  |
| **Cyclo(Leu-Gly)** | 0.352 |  |
| **Cyclo(Ala-Val)** | 0.342 |  |
| 2-Methylalanine | 0.138 |  |
| Propylene glycol | 1933.295 | Polyols |
| D-Fructose | 320.982 |  |
| D-(-)-Tagatose | 192.404 |  |
| Glycerol | 105.452 |  |
| D-Glucose | 103.602 |  |
| D-Glucitol | 94.352 |  |
| Aucubin | 85.102 |  |
| D-Erythro-pentopyranose, 2-deoxy- | 63.549 |  |
| Myo-Inositol | 60.126 |  |
| Erythritol | 56.889 |  |
| β-Lyxose | 30.711 |  |
| 2,3-Dihydroxy-2-methylpropanoic acid | 27.658 |  |
| D-(+)-Xylose | 21.923 |  |
| D-(-)-Ribofuranose | 18.223 |  |
| **D-Galactose** | 17.113 |  |
| Xylitol | 13.043 |  |
| Erythro-pentonic acid, 2-deoxy- | 12.58 |  |
| D-Erythrose | 10.175 |  |
| **Arabinose** | 9.343 |  |
| 2-Deoxy-d-arabino-hexono-1,4-lactone | 8.973 |  |
| **2,3,4,5-Tetrahydroxypentanoic acid-1,4-lactone** | 8.048 |  |
| 3-Hydroxy-2,3-dihydromaltol | 7.215 |  |
| **2-Deoxypentofuranose** | 6.753 |  |
| **Scyllo-Inositol** | 4.625 |  |
| **Glyceric acid** | 4.07 |  |
| 2,3,4-Trihydroxybutyric acid | 3.423 |  |
| 2,3-Butanediol | 3.073 |  |
| **Myo-Inositol, phosphate** | 3.053 |  |
| 2,3-Dihydroxy-2-methylbutanoic acid | 1.573 |  |
| **α-D-Mannose** | 0.925 |  |
| **Erythrono-1,4-lactone, (E)-** | 0.879 |  |
| **Caffeic acid** | 0.527 |  |
| **9,12-Octadecadienoic acid (Z,Z)-** | 3.423 | Fatty acids and its glycerids |
| Arachidic acid | 1.018 |  |
| **2,3-Dihydroxypropyl icosanoate** | 0.916 |  |
| Lactic Acid | 7057.913 | Hydroxy acids |
| Butanedioic acid, (E) | 169.279 |  |
| Tartaric acid | 53.559 |  |
| Hydracrylic acid | 6.845 |  |
| 2,4-Dihydroxybutanoic acid | 3.7 |  |
| **Glycolic acid** | 1.896 |  |
| Pentanedioic acid | 1.665 |  |
| 3-Phenyllactic acid | 26.271 | Aromatic compound |
| 4-Hydroxyphenyllactic acid | 12.95 |  |
| **Tyramine** | 0.833 |  |
| **Benzoic acid** | 0.749 |  |
| **5-Hydroxymethyl-2-furoic acid** | 0.546 |  |
| **4-Hydroxybenzoic acid** | 0.111 |  |
| Niacin | 1.295 | Nucleotides and other heterocyclic compound |
|  |  |  |
| 1-Hexadecanol | 199.805 | Others |

^1^ Metabolites that is bold are unique for *L.fermentum* U-21

**Supplementary Table 3.** Metabolites of *L.fermentum* U-21’s vesicles divided by main classes

| **Metabolite name^1^** | **Concentration, μg/g** | **Class** |
| --- | --- | --- |
| L-5-Oxoproline | 4.422 | Amino acids and peptides |
| **L-Glutamic acid** | 1.684 |  |
| L-Serine | 1.064 |  |
| L-Aspartic acid | 0.981 |  |
| Pyroglutamic acid | 0.851 |  |
| **L-Methionine** | 0.25 |  |
| **L-Threonine** | 0.231 |  |
| **Glycine** | 0.111 |  |
| **L-Valine** | 0.07 |  |
| **L-Norleucine** | 0.065 |  |
| L-Leucine | 0.041 |  |
| **L-Phenylalanine** | 0.022 |  |
| **Cyclo(Leu-Pro)]** | 0.004 |  |
| D-Glucose | 1.98 | Polyols |
| D-Fructose | 1.739 |  |
| **2,3,4,5-Tetrahydroxypentanoic acid-1,4-lactone** | 1.295 |  |
| Levoglucosan | 1.184 |  |
| **Erythro-pentonic acid, 2-deoxy-** | 0.981 |  |
| Myo-Inositol, phosphate | 0.638 |  |
| Xylitol | 0.611 |  |
| Arabinose | 0.537 |  |
| **2-Deoxy-d-arabino-hexono-1,4-lactone** | 0.435 |  |
| Myo-Inositol | 0.389 |  |
| **D-(+)-Gluconolactone** | 0.361 |  |
| D-Erythro-pentopyranose, 2-deoxy- | 0.231 |  |
| **Scyllo-Inositol** | 0.139 |  |
| Erythritol | 0.12 |  |
| **D-Gulose** | 0.12 |  |
| **Erythrono-1,4-lactone, (Z)-** | 0.12 |  |
| **D-Gluconic acid** | 0.102 |  |
| **Caffeic acid** | 0.009 |  |
| **D-Galactose** | 0.002 |  |
| **α-D-Mannose** | 0.002 |  |
| Myristic acid | 1.517 | Fatty acids and its glycerids |
| Heptadecanoic acid | 0.842 |  |
| 2-Monostearin | 0.777 |  |
| **10-Nonadecenoic acid, (Z)-** | 0.685 |  |
| **9-Octadecenoic acid, (Z)-** | 0.638 |  |
| 4-Octenoic acid | 0.611 |  |
| Arachidic acid | 0.416 |  |
| Heptadecanoic acid, glycerine-(1)-monoester | 0.333 |  |
| 2,3-Dihydroxypropyl icosanoate | 0.324 |  |
| **Octanoic acid** | 0.241 |  |
| **Nonanoic acid** | 0.222 |  |
| **Dodecanamide** | 0.019 |  |
| Lactic Acid | 3.589 | Hydroxy acids |
| Tartaric acid | 2.868 |  |
| Butanedioic acid, (E) | 1.943 |  |
| **β-Hydroxypyruvic acid** | 1.092 |  |
| **Glyoxylic acid** | 0.444 |  |
| **2-Butenedioic acid, (Z)-** | 0.13 |  |
| Glycolic acid | 0.086 |  |
| Protocatechoic acid | 1.175 | Aromatic compound |
| Benzoic acid | 0.379 |  |
| Benzoic acid, 4-ethoxy-, ethyl ester | 0.04 |  |
| **Vanillic Acid** | 0.031 |  |
| **Hydroquinone** | 0.011 |  |
| Benzaldehyde | 0.009 |  |
| **4-Hydroxybenzaldehyde** | 0.005 |  |
| **Phloretin** | 0.004 |  |
| **5-Methyluridine** | 0.463 | Nucleotides and other heterocyclic compound |
| 4,6-Diamino-5-formamidopyrimidine, N,N'- | 0.241 |  |
| **Uridine-5'-monophosphate** | 0.102 |  |
| **Adenine** | 0.032 |  |
| Niacin | 0.018 |  |
| **Octadecane** | 1.822 | Others |
| **Tetracosane** | 1.517 |  |
| Urea | 0.071 |  |
| **Aceturic acid** | 0.059 |  |
| Cyclohexaneacetic acid | 0.029 |  |

^1^ Metabolites that is bold are unique for *L.fermentum* U-21

**Supplementary Table 4.** Unique metabolites of *L.fermentum* U-21 in the vesicles

| **Metabolite name** | **Concentration, μg/g** |
| --- | --- |
| Octadecane | 1.822 |
| L-Glutamic acid | 1.684 |
| Tetracosane | 1.517 |
| 2,3,4,5-Tetrahydroxypentanoic acid-1,4-lactone | 1.295 |
| β-Hydroxypyruvic acid | 1.092 |
| Erythro-pentonic acid, 2-deoxy- | 0.981 |
| 10-Nonadecenoic acid, (Z)- | 0.685 |
| 9-Octadecenoic acid, (Z)- | 0.638 |
| 5-Methyluridine | 0.463 |
| Glyoxylic acid | 0.444 |
| 2-Deoxy-d-arabino-hexono-1,4-lactone | 0.435 |
| D-(+)-Gluconolactone | 0.361 |
| L-Methionine | 0.25 |
| Octanoic acid | 0.241 |
| L-Threonine | 0.231 |
| Nonanoic acid | 0.222 |
| Scyllo-Inositol | 0.139 |
| 2-Butenedioic acid, (Z)- | 0.13 |
| D-Gulose | 0.12 |
| Erythrono-1,4-lactone, (Z)- | 0.12 |
| Glycine | 0.111 |
| D-Gluconic acid | 0.102 |
| Uridine-5'-monophosphate | 0.102 |
| L-Valine | 0.07 |
| L-Norleucine | 0.065 |
| Aceturic acid | 0.059 |
| Adenine | 0.032 |
| Vanillic Acid | 0.031 |
| L-Phenylalanine | 0.022 |
| Dodecanamide | 0.019 |
| Hydroquinone | 0.011 |
| Caffeic acid | 0.009 |
| 4-Hydroxybenzaldehyde | 0.005 |
| Cyclo(Leu-Pro)] | 0.004 |
| Phloretin | 0.004 |
| D-Galactose | 0.002 |
| α-D-Mannose | 0.002 |

**Supplementary Table 5.** Unique metabolites of *L.fermentum* U-21 in the cultural fluid

| **Metabolite name** | **Concentration, μg/g** |
| --- | --- |
| D-Galactose | 17.113 |
| Arabinose | 9.343 |
| 2,3,4,5-Tetrahydroxypentanoic acid-1,4-lactone | 8.048 |
| 2-Deoxypentofuranose | 6.753 |
| Scyllo-Inositol | 4.625 |
| Glyceric acid | 4.07 |
| 9,12-Octadecadienoic acid (Z,Z)- | 3.423 |
| Myo-Inositol, phosphate | 3.053 |
| Glycolic acid | 1.896 |
| α-D-Mannose | 0.925 |
| 2,3-Dihydroxypropyl icosanoate | 0.916 |
| Erythrono-1,4-lactone, (E)- | 0.879 |
| Tyramine | 0.833 |
| Benzoic acid | 0.749 |
| 5-Hydroxymethyl-2-furoic acid | 0.546 |
| Caffeic acid | 0.527 |
| Cyclo(Leu-Gly) | 0.352 |
| Cyclo(Ala-Val) | 0.342 |
| 4-Hydroxybenzoic acid | 0.111 |

**Supplementary Table 6.** Homologous proteins for biosynthesis pathway of aucubin.

| ***L.fermentum* U-21 protein** | **Homologous protein** | **% of homology** | **Enzyme** |
| --- | --- | --- | --- |
| WP_021349464.1 | BAG27325.1 | 99.487 | acetyl-CoA acetyltransferase |
| WP_021349108.1 | BAG27043.1 | 99.743 | hydroxymethylglutaryl-CoA synthase |
| WP_102116070.1 | BAG26517.1 | 99.059 | hydroxymethylglutaryl-CoA reductase |
| WP_003683265.1 | BAG27528.1 | 99.357 | mevalonate kinase |
| WP_003683263.1 | BAG27530.1 | 100.0 | phosphomevalonate kinase |
| WP_003683264.1 | BAG27529.1 | 99.696 | mevalonate diphosphate decarboxylase |
| WP_024501015.1 | BAG27531.1 | 99.723 | isopentenyl diphosphate delta-isomerase |
| WP_003683815.1 | BAG27596.1 | 100.0 | geranyltranstransferase |
| WP_003682840.1 | WP_003682840.1 | 100 | geraniol 8-hydroxylase |
| WP_024500900.1 | WP_012391194.1 | 99.5 | iridoid synthase |
| WP_054173690.1 | WP_012390783.1 | 99.711 | 8-hydroxygeraniol oxidoreductase |
| WP_004562952.1 | WP_012391602.1 | 99.705 | 8-hydroxygeraniol dehydrogenase |
| WP_014562285.1 | WP_012390931.1 | 46.7 | iridoid oxidase |
| WP_003682323.1 | NP_217917.1 | 30 | hydrolyze geranyl diphosphate (GPP) |
| WP_024500874.1 | NP_216182.1 | 25.3 | 7-deoxyloganic acid hydroxylase |
| WP_003682130.1 | NP_217474.1 | 30 | 7-deoxyloganetic acid glucosyl transferase |
| WP_003682544.1 | NP_216179.1 | 30 | (S)-8-oxocitronellyl enol synthase |

*BAG and WP proteins belong *to L.fermentum* IFO 3956; NP proteins belong to *M.tuberculesis* H37Rv

**Supplementary Table 7.** Homologous proteins of *L.fermentum* strains for biosynthesis pathway of niacin.

| ***L.fermentum* U-21 protein** | **Another strain** | **% of homology** | **Enzyme** |
| --- | --- | --- | --- |
| WP_003686217.1 | *L.fermentum* D12 | 99 | nicotinate-nucleotide adenylyltransferase |
| WP_004562721.1 | *L.fermentum* F-6 | 100 | ammonia-dependent NAD(+) synthetase |
| WP_003683354.1 | *L.fermentum* URCS 11 | 98 | NAD(P)+ transhydrogenase |
| WP_003686313.1 | *L.fermentum* 01-B1 | 99 | NAD+ kinase |
| WP_003682880.1 | *L.fermentum* S13 | 94 | 5'-ribonucleotide phosphohydrolase  HAD (haloacid dehalogenase)-IIA family hydrolase  YutD family protein |
| WP_003686077.1 |  |  |  |
| WP_021350339.1 | *L.fermentum* CYLB55 | 99 | 5'-methylthioadenosine/adenosylhomocysteine nucleosidase |
| WP_004562720.1 | *L.fermentum* Lf1 | 100 | nicotinate phosphoribosyltransferase |
| WP_012391523.1 |  |  |  |
| WP_003683228.1 | *L.fermentum* SK152 | 99 | nicotinate phosphoribosyltransferase |
| WP_003685361.1 | *L.fermentum* F-6 | 99 | NAD+ diphosphatase |

**Supplementary Table 8.** Top-30 most presented proteins in extracellular vesicles of *L.fermentum* U-21 according to MS analysis.

| **Locus Tag** | **Protein ID** | **Protein name** | **riBAQ, %** |
| --- | --- | --- | --- |
| C0965_RS08020 | WP_226557692.1 | LYZ2 domain-containing protein | 12.45694 |
| C0965_RS08080 | WP_003684127.1 | DUF2922 domain-containing protein | 4.320349 |
| C0965_RS02375 | WP_012390875.1 | Glyceraldehyde-3-phosphate dehydrogenase | 4.226349 |
| C0965_RS08855 | WP_004562952.1 | Alcohol dehydrogenase | 3.849536 |
| C0965_RS03760 | WP_003682040.1 | D-2-hydroxyacid dehydrogenase | 3.520841 |
| C0965_RS01255 | WP_003683996.1 | Lipoprotein | 3.26767 |
| C0965_RS10235 | WP_012391767.1 | 6-phosphogluconate dehydrogenase, decarboxylating | 3.063428 |
| C0965_RS10165 | WP_014562712.1 | Probable phosphoketolase | 2.927808 |
| C0965_RS07720 | WP_012391508.1 | Hsp20/alpha crystallin family protein | 2.8736 |
| C0965_RS02390 | WP_012390876.1 | Enolase | 1.969941 |
| C0965_RS02490 | WP_259359323.1 | LysM peptidoglycan-binding domain-containing protein | 1.95778 |
| C0965_RS03200 | WP_003682865.1 | Competence protein ComGC | 1.877403 |
| C0965_RS08015 | WP_226557690.1 | NlpC/P60 domain-containing protein | 1.803461 |
| C0965_RS08610 | WP_004562971.1 | Diacetyl reductase [(S)-acetoin forming] | 1.742148 |
| C0965_RS08010 | WP_080558617.1 | Lipoprotein | 1.696711 |
| C0965_RS05070 | WP_003683180.1 | DNA-binding protein HU | 1.155023 |
| C0965_RS02380 | WP_003682636.1 | Phosphoglycerate kinase | 1.147308 |
| C0965_RS10570 | WP_102116119.1 | Peptidoglycan hydrolase | 1.101364 |
| C0965_RS03690 | WP_021349940.1 | Elongation factor Tu | 1.052171 |
| C0965_RS09010 | WP_021350088.1 | Universal stress protein | 1.034549 |
| C0965_RS08705 | WP_021349528.1 | 50S ribosomal protein L18 | 0.903578 |
| C0965_RS05060 | WP_003683184.1 | 30S ribosomal protein S1 | 0.895721 |
| C0965_RS08620 | WP_004562969.1 | 30S ribosomal protein S9 | 0.878667 |
| C0965_RS06605 | WP_014562491.1 | Alpha-ketoacid dehydrogenase subunit beta | 0.764344 |
| C0965_RS08790 | WP_004562959.1 | 30S ribosomal protein S10 | 0.742743 |
| C0965_RS02875 | WP_102116050.1 | 30S ribosomal protein S4 | 0.733647 |
| C0965_RS10230 | WP_003685507.1 | Glucose-6-phosphate 1-dehydrogenase | 0.727841 |
| C0965_RS08725 | WP_003681594.1 | 50S ribosomal protein L5 | 0.720349 |
| C0965_RS08805 | WP_003681563.1 | 30S ribosomal protein S12 | 0.69286 |
| C0965_RS08760 | WP_003681579.1 | 50S ribosomal protein L22 | 0.674486 |
| C0965_RS04210 | WP_003681957.1 | 30S ribosomal protein S2 | 0.632826 |
| … | … | … | … |
| C0965_RS00195 | WP_014562056.1 | ATP-dependent Clp protease ATP-binding subunit (ClpL) | 0.127083 |

* Proteins selected as *L.fermentum* U-21 vesicles’ biomarkers are underlined.
